# LMO2 and IL2RG synergize in thymocytes to mimic the evolution of SCID-X1 gene therapy-associated T-cell leukaemia

**DOI:** 10.1038/leu.2016.116

**Published:** 2016-06-03

**Authors:** K Ruggero, O Al-Assar, J S Chambers, R Codrington, T Brend, T H Rabbitts

**Affiliations:** 1 Weatherall Institute of Molecular Medicine, MRC Molecular Haematology Unit, University of Oxford, John Radcliffe Hospital, Oxford, UK; 2 ABeterno Technologies Ltd, Cambridge, UK

The SCID-X1 disease occurs in males that lack a functional X-linked gene encoding the interleukin 2 receptor subunit gamma (IL2RG) and thus are immuno-deficient (reviewed in Rochman *et al.*^1^). Gene therapy has been a success in curing SCID-X1 in patients receiving autologous CD34^+^-bone marrow cells infected with retroviruses expressing *IL2RG*. This treatment protocol has, however, produced adverse T-cell effects where clonal T-cell leukaemias arose, and four have insertional mutagenesis of the T-cell oncogene *LMO2*.^2, 3, 4, 5^ *LMO2* is a T-cell oncogene first discovered via chromosomal translocations in T-cell acute leukaemia (T-ALL) (reviewed in Chambers and Rabbitts^6^). It is unclear if the T-cell neoplasias in the SCID-X1 patients are simply due to insertional activation of the *LMO2* gene or reflect synergy between LMO2 and IL2RG.^7, 8, 9^ Further, the recurrent involvement of *LMO2* in SCID-X1 leukaemias is puzzling as other T-cell oncogenes (for example, *TAL1/SCL*, *HOX11* and *LYL1*) might equally have been targets. This suggests that specific properties of LMO2 *per se* are required in these adverse events. The oncogenic potential of IL2RG itself also remains controversial. Although it causes T-cell lymphomas in mice transplanted with virally transduced haematopoetic stem cells,^10^ other studies have indicated that *IL2RG* is not an oncogene.^11^ Here we provide evidence that synergy is required between LMO2 and IL2RG proteins specifically in the T-cell lineage to elicit neoplasias and that additional mutations are required such as *Notch1* mutations like those in human T-ALL.^12^

We made new two transgenic strains where *Lmo2* or *IL2RG* expression uses the *Lck*-promoter vector (summarised in Figure 1a) to express in thymus T cells. *Lck-Lmo2* transgene expression was confirmed using deep sequencing RNA-seq (Ruggero and Rabbitts, manuscript in preparation), and expression of the *IL2RG* transgene was confirmed by flow cytometry (Supplementary Figure 1a). *Lck-Lmo2* expression causes a T-cell differentiation blockage at the CD4; CD8 double-negative stage (DN cells) (Supplementary Figure 1d) as first shown with *CD2-Lmo2* mice.^13, 14^ Expression of the Lck-IL2RG transgene alone did not affect the phenotype of the thymus population (Supplementary Figure 1c).

Double transgenic mice expressing both *LMO2* and *IL2RG* were generated by interbreeding. Both single *Lck-Lmo2* and double *Lck-Lmo2*; *Lck-IL2RG* transgenic lines develop clonal T-cell neoplasias characterised by thymoma and splenomegaly, after a latency period of several months. Kaplan–Meier survival curves are shown in Figure 1b. We note a significant difference in the time of appearance of T-cell thymomas in the *Lck-Lmo2* mice (mean occurrence of 320 days) compared with the double transgenic *Lck-Lmo2*; *Lck-IL2RG* counterparts (mean occurrence of 220 days). The *Lck-IL2RG* single transgenic mice did not show signs of ill health or alteration of thymocyte differentiation. Histological analysis of the single and double transgenic mouse tumours showed similar pathology with homogeneous cellularity in the thymus, loss of distinct red and white pulp in the spleen, and perivascular deposits in pale liver and kidneys (Supplementary Figures S2 and S3). Thus, thymic co-expression of LMO2 and IL2RG proteins synergistically accelerates the rate of T-cell tumour formation compared with LMO2 alone.

T-cell tumours in *Lck-Lmo2* and *Lck-Lmo2*; *Lck-IL2RG* mice were clonal as judged by Southern hybridisation analysis of T-cell receptor β-chain (*Tcrb*) gene rearrangement in thymoma genomic DNA (Supplementary Figures 4a and b). Most of the tumours analysed display rearrangement of one or both *Tcrb* alleles. The DN surface phenotype of the asymptomatic mice was essentially the same between strains, and the tumours were transplantable. A representative single *Lck-Lmo2* mouse (STG21) and two double transgenic mice (DTG21 and DTG22) were compared in more detail by amplification of unique genomic polymerase chain reaction (PCR) products using Vβ and Jβ PCR primers (Supplementary Figure 4c) demonstrating bi-allelic *Tcrb* rearrangements. STG21 has a DN2 phenotype (Figure 1d) similar to the *CD2-LMO2* strain.^13, 14, 15^ The two double transgenic *Lck-Lmo2*; *Lck-IL2RG* mice (DTG21 and DTG22, Figures 1e and f) had either a mixed DN1 and DN2 phenotype or a DN3 phenotype.

The long latency of tumours in the transgenic mice shows that LMO2 is necessary, but not sufficient for T-cell neoplasia and other genetic or epigenetic changes must occur for overt neoplasia. Furthermore, the decrease in the length of this asymptomatic period in *Lck-Lmo2*; *Lck-IL2RG* mice indicates the accumulation of these mutations occurs more rapidly and/or different mutations account for the rate increase. A number of mutations have been identified in LMO2-associated human T-ALL, including *NOTCH1* mutations that appear in nearly half of cases.^12^ We analysed the *Notch1* gene in the transgenic tumours and found mutation in 65 and 55% of samples, respectively, of the single and double transgenic tumours. Two types of mutation were observed (Figure 2). A hot spot for mutations was identified in exons 26–27 (Figure 2a) that code for the heterodimerisation domain, responsible for the non-covalent interaction between the trans-activator and the extracellular domains. A second set of common mutations were insertions in exon 34 coding for the proline, glutamic acid, serine, threonine-rich sequence (PEST) domain (Figure 2b). This region of the Notch1 protein regulates degradation and mutations, therein affecting the protein half-life.^12^ The mutations observed in our mouse T-cell neoplasias (both the *Lck-Lmo2* and *Lck-Lmo2*; *Lck-IL2RG* lines) faithfully recapitulate the mutations observed in human T-ALL.^12^ Thus, transit from asymptomatic and differentiation-blocked thymocytes to clonal neoplasia results from *Notch1* gene mutation in at least half of the cases.

In establishing the dual transgenic model of *LMO2* and *IL2RG*, we sought to elucidate the roles of these two genes in the adverse effects encountered in patients receiving SCID-X1 gene therapy. Our data show that *IL2RG* is not directly oncogenic in T cells. However, LMO2 and IL2RG functionally co-operate in thymocytes to accelerate tumourigenesis as *IL2RG* shortens the period in which cells acquire mutations required to drive leukaemia. Most likely, because LMO2 invokes thymocytes differentiation block at DN2/DN3 stages where IL2RA (CD25) is expressed, linking its effect to the other IL2R chain, IL2RG. It is intriguing as IL2RG is required to form a higher affinity IL2 receptor with IL2RA, whose downstream signalling affects T-cell proliferation and differentiation.^1^ The LMO2 and IL2RG co-operation that causes the faster tumour aetiology may be a consequence of LMO2-induced blockade at DN2/3 and expression of transgenic IL2RG resulting in IL2 receptor precluding these cells entering a prolonged resting state. Finally, it is noteworthy that not all SCID-X1 gene therapy patients developed leukaemia, although some had clones with detectable retroviral insertion at *LMO2*.^16^ This is presumably because LMO2 is not sufficient for T-cell oncogenesis^14^ and secondary mutations are needed for overt disease, such as *Notch1*. In our transgenic model, the high penetrance of T-cell neoplasia reflects the high number of T cells available for random secondary mutations to occur.

In our model, ectopic expression of *LMO2* and *IL2RG* is restricted to the T-cell lineage, establishing that co-expression of these genes in SCID-X1 therapy patients is sufficient to drive development of leukaemia. Although SCID-X1 patients with the requisite retroviral insertion may co-express *LMO2* and *IL2RG* in bone marrow pluripotent cells, it seems likely that leukaemogenesis is only initiated once those cells arrive in the thymus to be subjected to the *LMO2*-mediated differentiation block. Thus, *LMO2* gene expression in bone marrow progenitors is not relevant *per se*, but rather the probability of inserting into any open chromatin gene is related to the dose of transducing retrovirus and the number of CD34^+^ HSC transduced for the gene therapy.^7^ In Wiskott–Aldrich gene therapy, patients also developed leukaemias showing *LMO2* activation through retroviral insertion.^17^ Interestingly, several of these leukaemias had secondary insertions at the *TAL1* gene, known to enhance tumourigenesis in mice,^14^ or the *LYL1* gene encoding another LMO2 interaction partner.^18^

Correction of the IL2RG deficiency in SCID-X1 is a significant success that was tempered when patients developed therapy-related T-cell leukaemia. It is now clear that the adverse T-cell effects in the patients resulted from an unpredicted consequence of insertional mutagenesis into a gene (*LMO2*) that has the power, when aberrantly activated, to affect the differentiation of the very cells that are defective in SCID-X1.

## Figures and Tables

**Figure 1:**
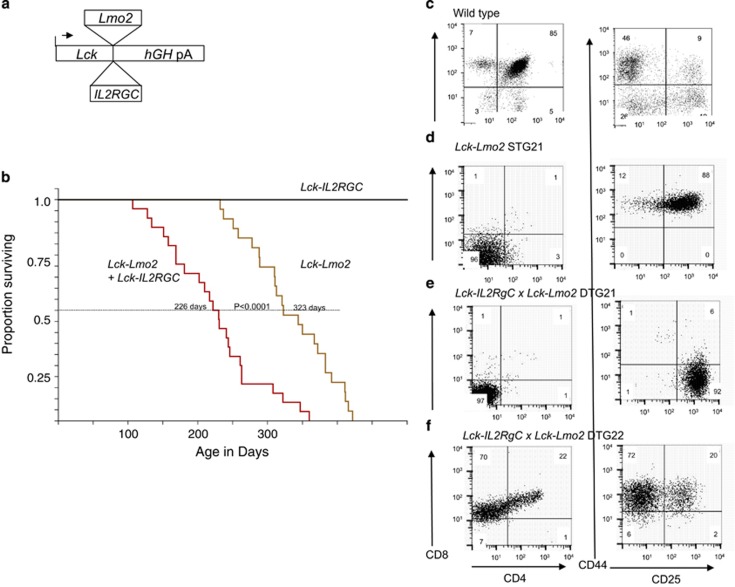
T-cell tumours in *Lck-Lmo2* and *Lck-Lmo2*; *Lck-IL2RG* transgenic mice. (**a**) Transgenic mice were made expressing Lmo2 and/or IL2RG in thymocytes. Cohorts of mice expressing either *LMO2* or *IL2RG* or both transgenes were monitored over an 18-month period, and disease assessed by macroscopic changes to habit and subsequent post-mortem. (**b**) Kaplan–Meier comparison of disease incidence. The *Lmo2* and *IL2RG* double transgenic (*n*=21) group developed T-cell neoplasia at an accelerated rate compared with single *Lmo2* transgenic mice (*P*-value <0.0001) (*n*=22). Single *IL2RG* mice did not develop tumours (*n*=22). (**c**-**f**) Flow cytometry of CD4, CD8, CD25 and CD44 of a wild-type C57Bl6 mouse (male; 21 weeks) (**c**) secondary tumours derived from the spleen of *Rag1* null recipient mice transplanted with *Lck-Lmo2* STG1 (**d**) *Lck-Lmo2*; *Lck-IL2RG* DTG21 (**e**) or *Lck-Lmo2*; *Lck-IL2RG* DTG22 (**f**) primary tumours. Cells were gated on CD90.2-positive population.

**Figure 2:**
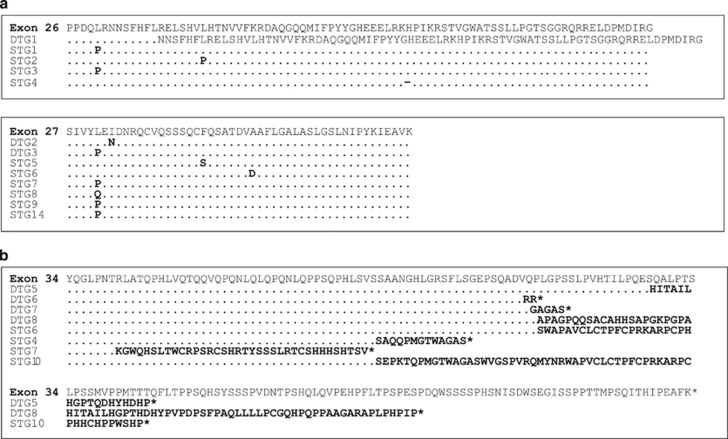
*Notch1* mutations in transgenic tumours. Genomic DNA from thymoma samples were analysed by MCA for potential *Notch1* mutations at exon 26, 27 and 34 regions. Positive samples were sequenced across the appropriate exon regions. Mutated amino acids are shown in bold. Point mutations occur in exons 26 and 27 (**a**), except one *Lck-Lmo2*; *Lck-IL2RG* tumour that has a frame shift in exon 26. Exon 34 (**b**) changes result in frame shifts.
