# Supplementary material for: LMO2 and IL2RG synergize in thymocytes to mimic the evolution of SCID-X1 gene therapy-associated T-cell leukaemia
Source: Leukemia. 2016 Jun 3;30(9):1959–62. doi: 10.1038/leu.2016.116 (PMC5227057; doi:10.1038/leu.2016.116)
Supplement: Supplementary Information [file leu2016116x1.doc]

**Supplementary Information**

**Materials and Methods**

**Mouse strains**

Mouse *Lmo2* or human *IL2RG* cDNAs were cloned into the BamHI site of the *pLck-hGHpA* vector. The sequences of *IL2RG* and of *Lmo2* used are shown in Supplementary Figures S5 and S6. Linearized plasmid fragments were injected into CBA/C57Bl/6 fertilized egg pro-nuclei and transgenic mice generated. F1 mice were identified using a 5’ Lck probe in Southern blotting and these were bred with C57Bl/6 mice. Subsequent generations were inter-bred to achieve homozygosity. Inter-breeding of homozygous *Lck-Lmo2* and *Lck-IL2RG* produced heterozygous double transgenic offspring.

Secondary tumours were generated by intra-venous transplantation of a bolus of 10^6^ donor tumour cells into homozygous *Rag1* null recipient mice.

**Gene expression profiling**

Thymus biopsies from *Lck-Lmo2* and wild type mice were crushed through a 70 µm Cell Strainer to obtain single cell suspensions. DN2 and DN3 cells were sorted using a Beckman Coulter MoFlo Cell Sorter or a Becton Dickinson FACSAria III Sorter, on the basis of the following combination of surface markers: CD90+CD4-CD8-CD25+CD44+ (DN2) and CD90+CD4-CD8-CD25+CD44- (DN3). RNA was extracted using the RNeasy micro kit (Qiagen) and the concentration and quality was assessed with an Agilent RNA 6000 Pico Assay on Agilent 2100 Bioanalyzer. Sequencing library generation and sequencing were performed by the High-Throughput Genomics Group at the Oxford Wellcome Trust Centre for Human Genetics (Ruggero & Rabbitts, manuscript in preparation).

**Histology and flow cytometry analysis**

Animals were monitored frequently for signs of ill health. At that point, humane sacrifice was carried out and macroscopic examination conducted. Tissues were excised and fixed in 4% formalin before processing. Single cell suspensions of thymus and spleen cells were made from segments of unfixed tissue and either nucleic acids prepared or surface marker analysis was carried out by flow cytometry using either Beckman Coulter LSRII or CyAn™ ADP Analyzers. The antibodies used in the study were purchased from BD Biosciences (PE-Cy™7 Rat Anti-Mouse CD4: 561099, FITC Rat Anti-Mouse CD8a: 553030, V450 Rat Anti-Mouse CD90.2: 561643, APC Rat Anti-Mouse CD25: 561048, PE Rat Anti-Mouse CD44: 553134).

Histology was performed on wax embedded formalin fixed tissues using haematoxylin and eosin staining of 20uM sections.

***Notch1* mutation analysis**

Melting curve analysis (MCA) of exons 26, 27 and 34 was carried out on all tumour DNA samples using the primer pairs below to generate DNA for MCA. PCR products with evidence of mutation(s) were subject to DNA sequencing for confirmation.

| Exon | Size (bp) | Forward primer | Reverse |
| --- | --- | --- | --- |
| 26 | 498 | ccggatgggtttgaggtccacagg | gaaacagcccacaacagcag |
| 27 | 299 | ggctagggagtcagagctggt | tgaacccttgtcctctgcaa |
| 34 | 593 | gcagcctctccaccaatacc | tcctgcatcccacatctctg |

***Tcrb* gene analysis**

Clonality was established either using HindIII-digested tumour DNA using Southern hybridization and a *Tcrb* *Jβ2* as described [1] or by genomic PCR of *Tcrb*. Genomic DNA was extracted for PCR with AllPrep DNA/RNA Mini Kit (Qiagen). Splenocytes from a wild type animal were used as control samples. *Tcrb* was amplified with forward Vβ5 or Vβ14 and reverse Jβ2.7 or Jβ1.7 primers (primers sequences from [2] using a protocol modified from [3]. 50ng of genomic DNA was amplified with Phusion High-Fidelity DNA polymerase (New England Biolabs) (98° 40s, 34 cycles 98° 10s, 64° 10s, 72° 15s, extension for 5 min at 72°). PCR products were fractionated on 1.5% agarose gels.

Vβ5 GCCGCCAGAGCTCATGTTTC

Vβ14 GCTGGAGTCACCCAGTCTC

Jβ2.7 TGAGAGCTGTCTCCTACTATCGATT

Jβ1.7 CCAAGACCATGGTCATCCAAC

**References**

1. LeFranc, M.P., A. Forster, R. Baer, M.A. Stinson, and T.H. Rabbitts, Diversity and rearrangement of the human T cell rearranging gamma genes: nine germ-line variable genes belonging to two subgroups. Cell, **45**: 1986; 237-46.

2. Martins, V.C., K. Busch, D. Juraeva, C. Blum, C. Ludwig, V. Rasche, F. Lasitschka, S.E. Mastitsky, B. Brors, T. Hielscher, H.J. Fehling, and H.R. Rodewald, Cell competition is a tumour suppressor mechanism in the thymus. Nature, **509**: 2014; 465-70.

3. Gerby, B., C.S. Tremblay, M. Tremblay, S. Rojas-Sutterlin, S. Herblot, J. Hebert, G. Sauvageau, S. Lemieux, E. Lecuyer, D.F. Veiga, and T. Hoang, SCL, LMO1 and Notch1 Reprogram Thymocytes into Self-Renewing Cells. PLoS Genet, **10**: 2014; e1004768.
