# Supplementary material for: LMO2 and IL2RG synergize in thymocytes to mimic the evolution of SCID-X1 gene therapy-associated T-cell leukaemia
Source: Leukemia. 2016 Jun 3;30(9):1959–62. doi: 10.1038/leu.2016.116 (PMC5227057; doi:10.1038/leu.2016.116)
Supplement: Supplementary Figures [file leu2016116x2.pdf]

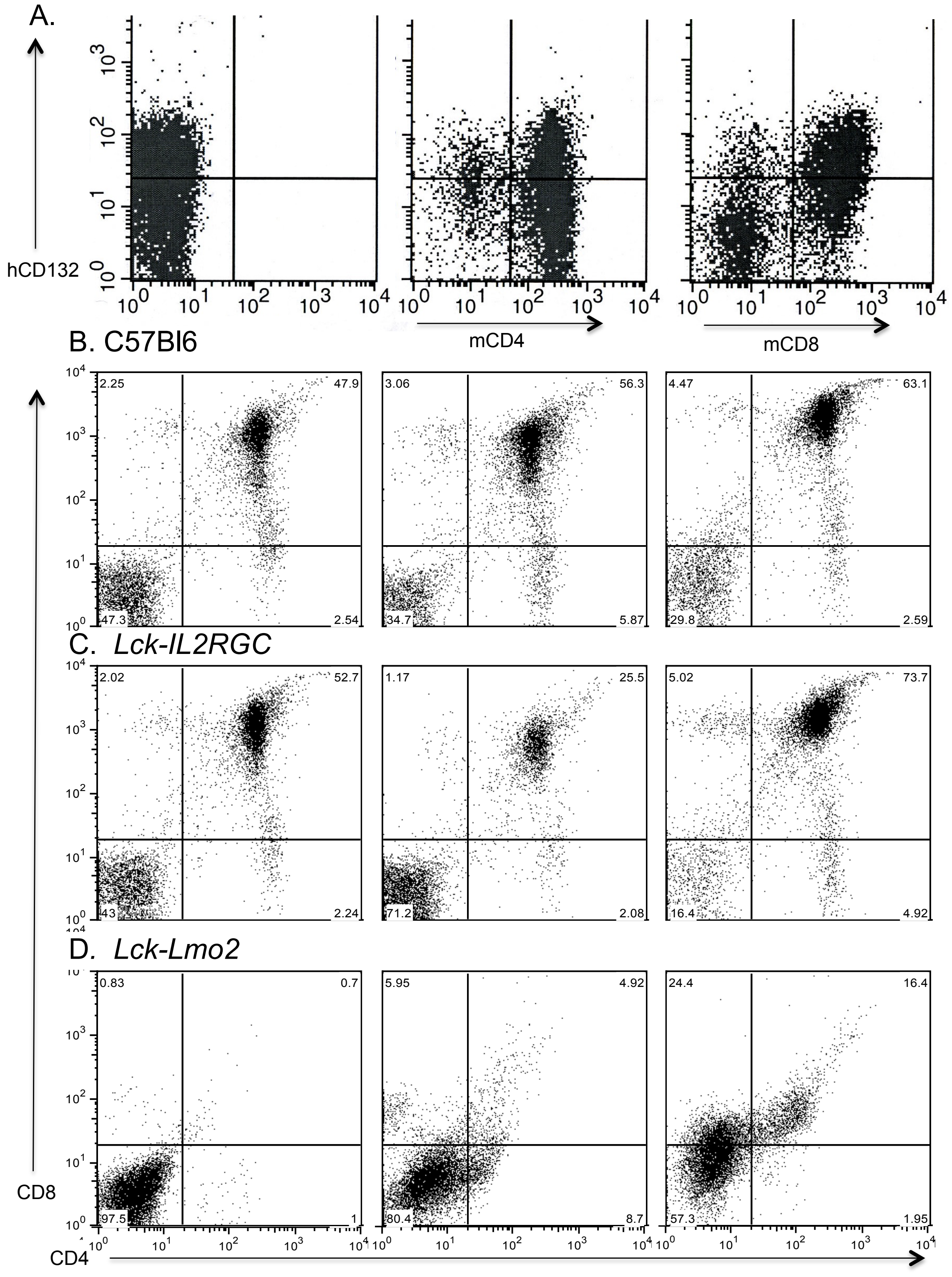

Figure S1. Flow cytometry of thymocytes from *Lck-Lmo2* and *Lck-IL2RG* transgenic mice

A. Expression of the human IL2RG transgenic protein

Thymocytes were prepared from a 5 week old *Lck-IL2RG* mouse, stained with anti-human CD132-PE and either anti-mouse CD4-FITC or anti-mouse CD8-FITC and analyzed by FACS.

B-D. Comparison of thymus T cell differentiation profiles of wild-type (B), *Lck-IL2RG* (C) and *Lck-Lmo2* (D) mice at approximately 20 weeks using anti-CD8-FITC and anti-CD4-PE coupled antibodies. Three individual mice are shown for comparison.

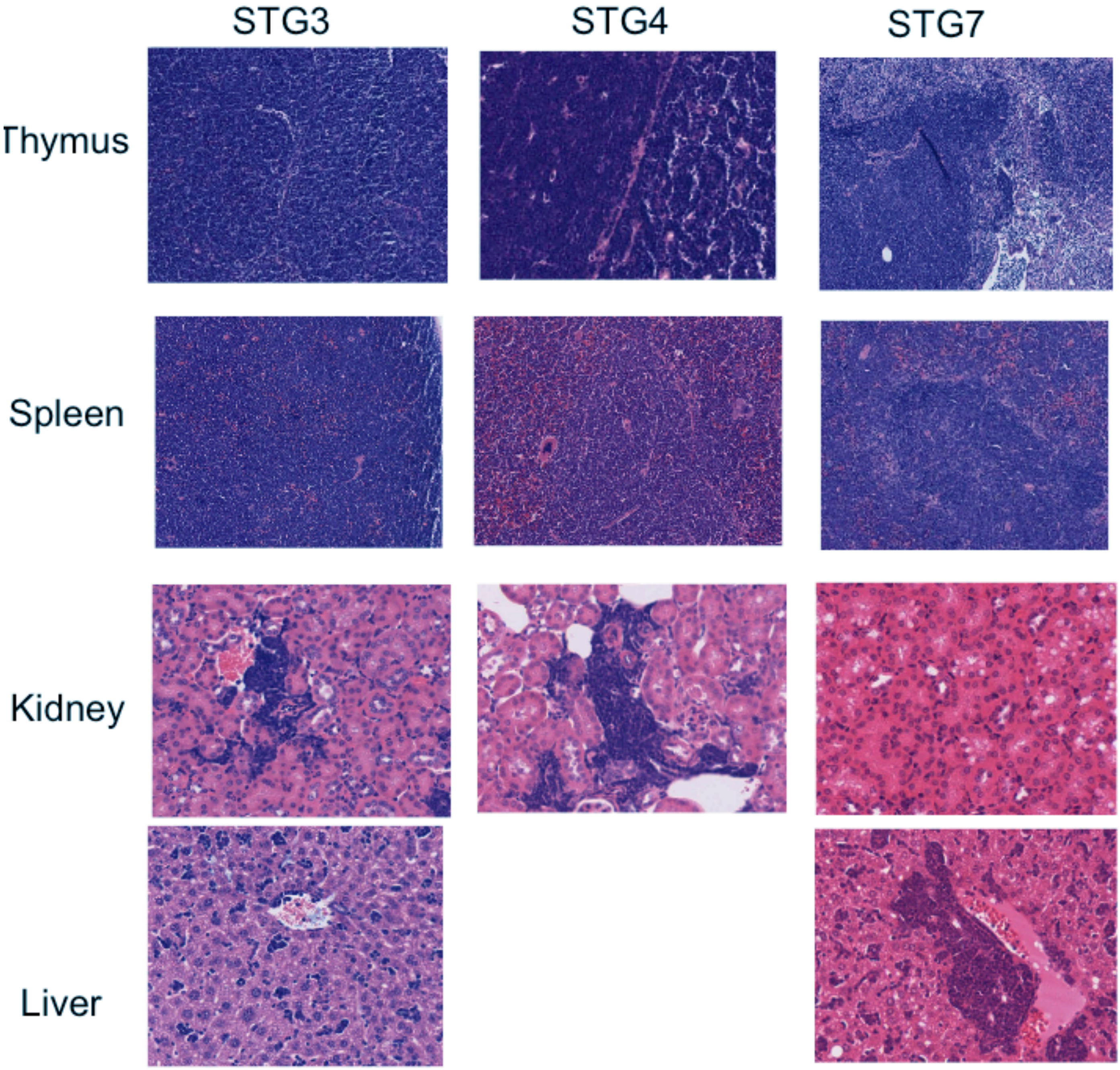

Figure S2. Histopathology of tissues from *Lck-Lmo2* tumour-bearing transgenic mice
At the time of sacrifice tissues were dissected and transferred to 4% formalin for fixation. Wax embedded sections were made, stained using haematoxylin and eosin and photographed at 40X magnification.

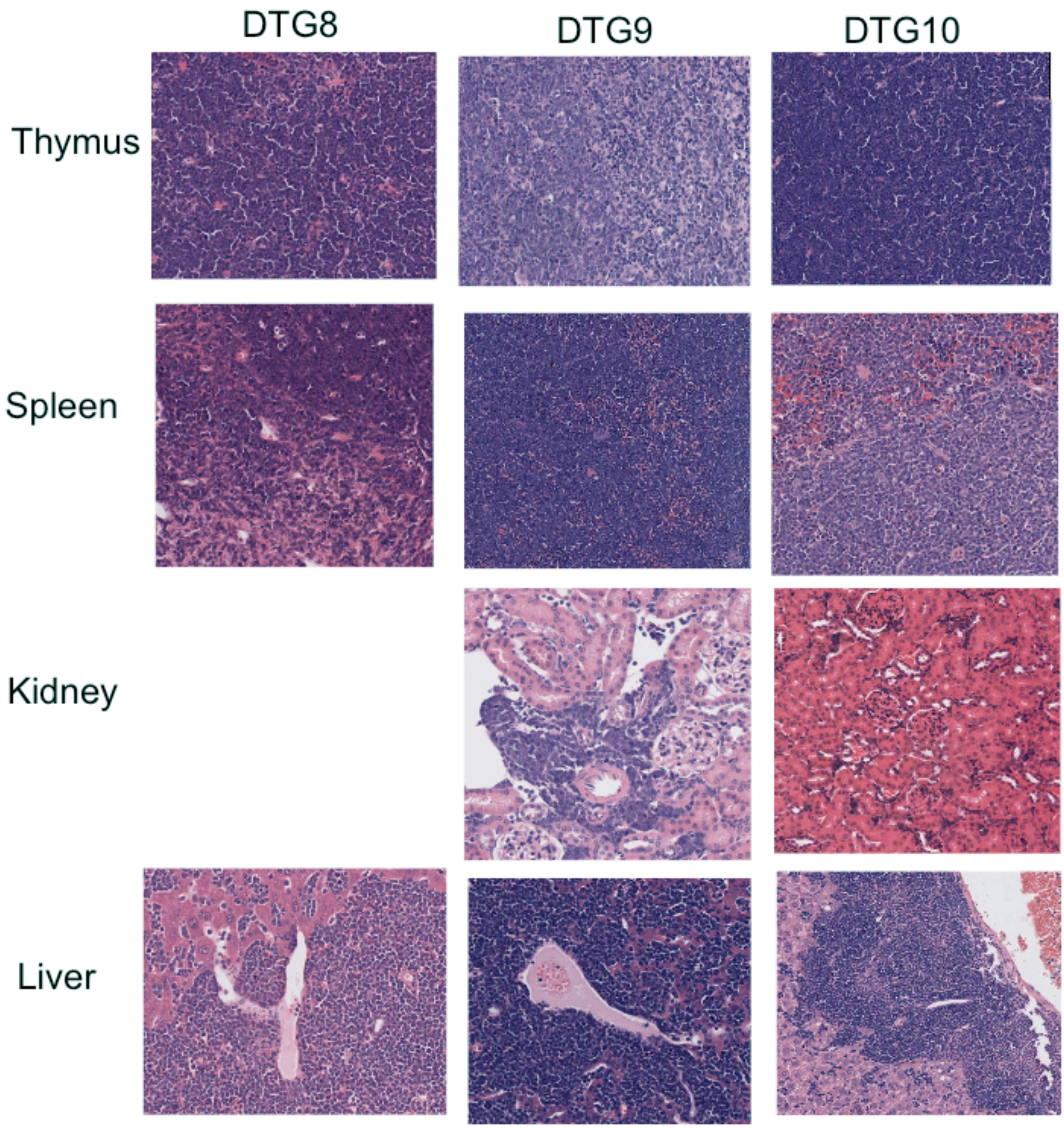

Figure S3. Histopathology of tissues from *Lck-Lmo2; Lck-IL2RG* tumour-bearing transgenic mice
At the time of sacrifice tissues were dissected and transferred to 4% formalin for fixation. Wax embedded sections were made, stained using haematoxylin and eosin and photographed at 40X magnification.

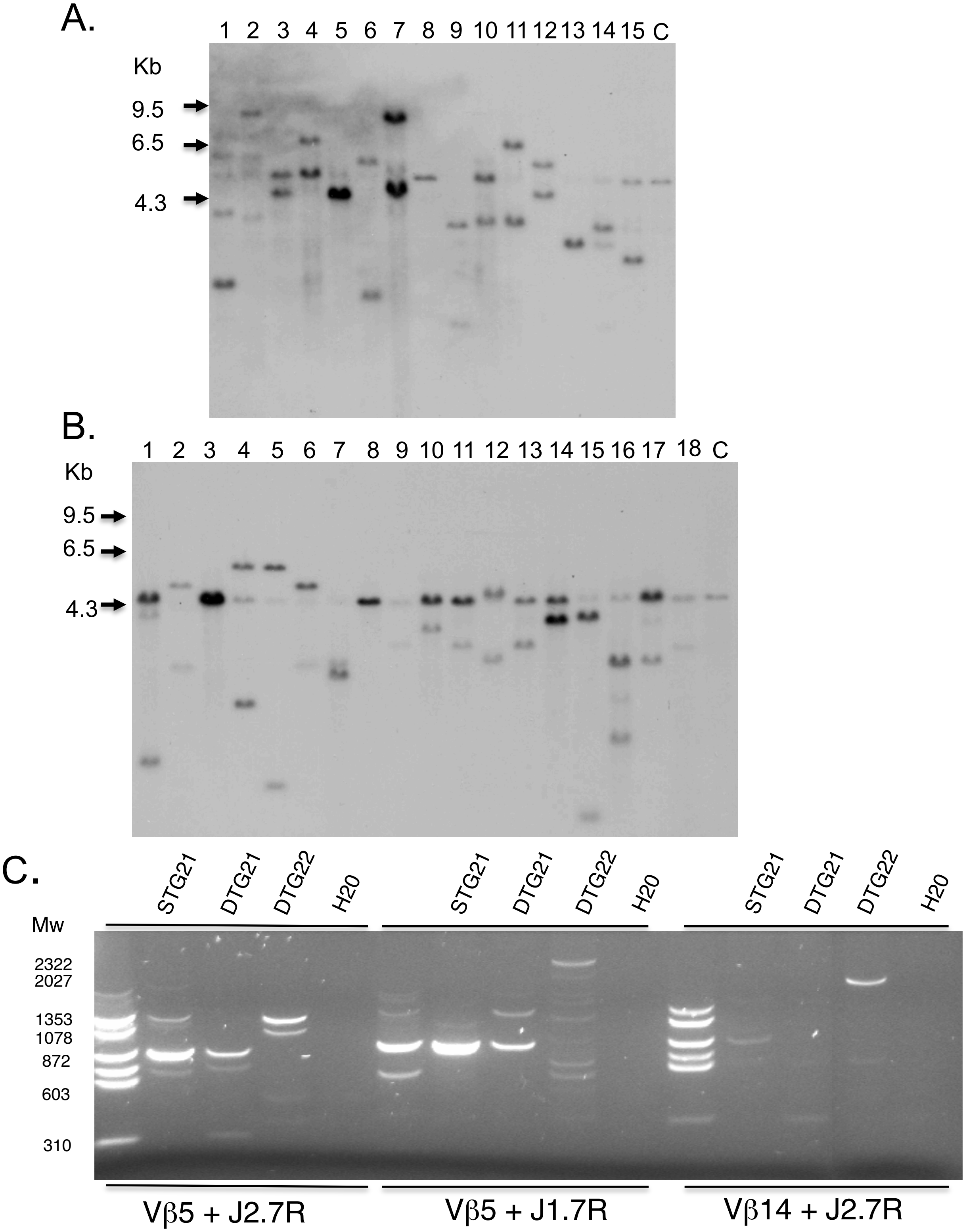

Supplementary figure S4. Clonality of LMO2 & LMO2;IL2RG transgenic mouse tumours

Genomic DNA was prepared from primary tumours of single and double transgenic mice. Clonality was assessed by Southern hybridization using a TCR Cβ1 probe (**A, B**). The numbers refer to tumour numbers and C = wild type mouse tail DNA. The size markers are from λ DNA digested with HindIII.

C. Genomic DNA PCR of a wild type (wt) spleen, or tumour DNA of *Lck-Lmo2* (STG22), *Lck-Lmo2; Lck-IL2RG* (DTG21) or DTG22 mice. Genomic PCR was carried out using forward Vβ5 or Vβ14 and reverse Jβ2.7 or Jβ1.7 primers and products fractionated on agarose gels.

Size markers are from ϕX174 DNA digested with HaeIII.

Figure S5. cDNA sequence of human ILRG cloned into Lck-promoter cassette

```
         10        20        30        40        50        60
GAAGAGCAAGCGCCATGTTGAAGCCATCATTACCATTCACATCCCTCTTATTCCTGCAGC
CTTCTCGTTCGCGGTACAACTTCGGTAGTAATGGTAAGTGTAGGGAGAATAAGGACGTCG
               M  L  K  P  S  L  P  F  T  S  L  L  F  L  Q>

         70        80        90       100       110       120
TGCCCCTGCTGGGAGTGGGGCTGAACACGACAATTCTGACGCCCAATGGGAATGAAGACA
ACGGGGACGACCCTCACCCCGACTTGTGCTGTTAAGACTGCGGGTTACCCTTACTTCTGT
L  P  L  L  G  V  G  L  N  T  T  I  L  T  P  N  G  N  E  D>

        130       140       150       160       170       180
CCACAGCTGATTTCTTCCTGACCACTATGCCCACTGACTCCCTCAGTGTTTCCACTCTGC
GGTGTCGACTAAAGAAGGACTGGTGATACGGGTGACTGAGGGAGTCACAAAGGTGAGACG
T  T  A  D  F  F  L  T  T  M  P  T  D  S  L  S  V  S  T  L>

        190       200       210       220       230       240
CCCTCCCAGAGGTTCAGTGTTTTGTGTTCAATGTCGAGTACATGAATTGCACTTGGAACA
GGGAGGGTCTCCAAGTCACAAAACACAAGTTACAGCTCATGTACTTAACGTGAACCTTGT
P  L  P  E  V  Q  C  F  V  F  N  V  E  Y  M  N  C  T  W  N>

        250       260       270       280       290       300
GCAGCTCTGAGCCCCAGCCTACCAACCTCACTCTGCATTATTGGTACAAGAACTCGGATA
CGTCGAGACTCGGGGTCGGATGGTTGGAGTGAGACGTAATAACCATGTTCTTGAGCCTAT
S  S  S  E  P  Q  P  T  N  L  T  L  H  Y  W  Y  K  N  S  D>

        310       320       330       340       350       360
ATGATAAAGTCCAGAAGTGCAGCCACTATCTATTCTCTGAAGAAATCACTTCTGGCTGTC
TACTATTTCAGGTCTTCACGTCGGTGATAGATAAGAGACTTCTTTAGTGAAGACCGACAG
N  D  K  V  Q  K  C  S  H  Y  L  F  S  E  E  I  T  S  G  C>

        370       380       390       400       410       420
AGTTGCAAAAAAAGGAGATCCACCTCTACCAAACATTTGTTGTTCAGCTCCAGGACCCAC
TCAACGTTTTTTTCCTCTAGGTGGAGATGGTTTGTAAACAACAAGTCGAGGTCCTGGGTG
Q  L  Q  K  K  E  I  H  L  Y  Q  T  F  V  V  Q  L  Q  D  P>

        430       440       450       460       470       480
GGGAACCCAGGAGACAGGCCACACAGATGCTAAAACTGCAGAATCTGGTGATCCCCTGGG
CCCTTGGGTCCTCTGTCCGGTGTGTCTACGATTTTGACGTCTTAGACCACTAGGGGACCC
R  E  P  R  R  Q  A  T  Q  M  L  K  L  Q  N  L  V  I  P  W>

        490       500       510       520       530       540
CTCCAGAGAACCTAACACTTCACAAACTGAGTGAATCCCAGCTAGAACTGAACTGGAACA
GAGGTCTCTTGGATTGTGAAGTGTTTGACTCACTTAGGGTCGATCTTGACTTGACCTTGT
A  P  E  N  L  T  L  H  K  L  S  E  S  Q  L  E  L  N  W  N>

        550       560       570       580       590       600
ACAGATTCTTGAACCACTGTTTGGAGCACTTGGTGCAGTACCGGACTGACTGGGACCACA
TGTCTAAGAACTTGGTGACAAACCTCGTGAACCACGTCATGGCCTGACTGACCCTGGTGT
N  R  F  L  N  H  C  L  E  H  L  V  Q  Y  R  T  D  W  D  H>

        610       620       630       640       650       660
GCTGGACTGAACAATCAGTGGATTATAGACATAAGTTCTCCTTGCCTAGTGTGGATGGGC
CGACCTGACTTGTTAGTCACCTAATATCTGTATTCAAGAGGAACGGATCACACCTACCCG
S  W  T  E  Q  S  V  D  Y  R  H  K  F  S  L  P  S  V  D  G>

        670       680       690       700       710       720
AGAAACGCTACACGTTTCGTGTTCGGAGCCGCTTTAACCCACTCTGTGGAAGTGCTCAGC
TCTTTGCGATGTGCAAAGCACAAGCCTCGGCGAAATTGGGTGAGACACCTTCACGAGTCG
Q  K  R  Y  T  F  R  V  R  S  R  F  N  P  L  C  G  S  A  Q>
```

```
       730        740        750        760        770        780
ATTGGAGTGAATGGAGCCACCCAATCCACTGGGGGAGCAATACTTCAAAAGAGAATCCTT
TAACCTCACTTACCTCGGTGGGTTAGGTGACCCCCTCGTTATGAAGTTTTCTCTTAGGAA
H  W  S  E  W  S  H  P  I  H  W  G  S  N  T  S  K  E  N  P>

       790        800        810        820        830        840
TCCTGTTTGCATTGGAAGCCGTGGTTATCTCTGTTGGCTCCATGGGATTGATTATCAGCC
AGGACAAACGTAACCTTCGGCACCAATAGAGACAACCGAGGTACCCTAACTAATAGTCGG
F  L  F  A  L  E  A  V  V  I  S  V  G  S  M  G  L  I  I  S>

       850        860        870        880        890        900
TTCTCTGTGTGTATTTCTGGCTGGAACGGACGATGCCCCGAATTCCCACCCTGAAGAACC
AAGAGACACACATAAAGACCGACCTTGCCTGCTACGGGGCTTAAGGGTGGGACTTCTTGG
L  L  C  V  Y  F  W  L  E  R  T  M  P  R  I  P  T  L  K  N>

       910        920        930        940        950        960
TAGAGGATCTTGTTACTGAATACCACGGGAACTTTTCGGCCTGGAGTGGTGTGTCTAAGG
ATCTCCTAGAACAATGACTTATGGTGCCCTTGAAAAGCCGGACCTCACCACACAGATTCC
L  E  D  L  V  T  E  Y  H  G  N  F  S  A  W  S  G  V  S  K>

       970        980        990       1000       1010       1020
GACTGGCTGAGAGTCTGCAGCCAGACTACAGTGAACGACTCTGCCTCGTCAGTGAGATTC
CTGACCGACTCTCAGACGTCGGTCTGATGTCACTTGCTGAGACGGAGCAGTCACTCTAAG
G  L  A  E  S  L  Q  P  D  Y  S  E  R  L  C  L  V  S  E  I>

      1030       1040       1050       1060       1070       1080
CCCCAAAAGGAGGGGCCCTTGGGGAGGGGCCTGGGGCCTCCCCATGCAACCAGCATAGCC
GGGGTTTTCCTCCCCGGGAACCCCTCCCCGGACCCCGGAGGGGTACGTTGGTCGTATCGG
P  P  K  G  G  A  L  G  E  G  P  G  A  S  P  C  N  Q  H  S>

      1090       1100       1110       1120
CCTACTGGGCCCCCCCATGTTACACCCTAAAGCCTGAAACCTGA
GGATGACCCGGGGGGGTACAATGTGGGATTTCGGACTTTGGACT
P  Y  W  A  P  P  C  Y  T  L  K  P  E  T  *>
```

Figure S6. cDNA sequence of mouse Lmo2 cloned into Lck-promoter cassette

```
        10        20        30        40        50
GCCCCCCTCCCCAATGTCCTCGGCCATCGAAAGGAAGAGCCTGGACCCGT
CGGGGGGAGGGGTTACAGGAGCCGGTAGCTTTCCTTCTCGGACCTGGGCA
              M  S  S  A  I  E  R  K  S  L  D  P>

        60        70        80        90       100
CTGAGGAACCCGTGGATGAGGTGCTGCAGATACCCCCATCCCTGCTGACA
GACTCCTTGGGCACCTACTCCACGACGTCTATGGGGGTAGGGACGACTGT
S  E  E  P  V  D  E  V  L  Q  I  P  P  S  L  L  T>

       110       120       130       140       150
TGTGGTGGCTGCCAGCAGAACATAGGGGACCGCTACTTCCTGAAAGCCAT
ACACCACCGACGGTCGTCTTGTATCCCCTGGCGATGAAGGACTTTCGGTA
 C  G  G  C  Q  Q  N  I  G  D  R  Y  F  L  K  A  I>

       160       170       180       190       200
CGACCAGTACTGGCATGAGGATTGCCTCAGCTGTGACCTCTGTGGGTGTC
GCTGGTCATGACCGTACTCCTAACGGAGTCGACACTGGAGACACCCACAG
  D  Q  Y  W  H  E  D  C  L  S  C  D  L  C  G  C>

       210       220       230       240       250
GGCTGGGAGAGGTGGGGAGGCGCCTCTACTACAAGCTGGGACGGAAATTG
CCGACCCTCTCCACCCCTCCGCGGAGATGATGTTCGACCCTGCCTTTAAC
R  L  G  E  V  G  R  R  L  Y  Y  K  L  G  R  K  L>

       260       270       280       290       300
TGCAGGAGAGACTATCTCAGGCTTTTTGGTCAGGATGGTCTCTGTGCATC
ACGTCCTCTCTGATAGAGTCCGAAAAACCAGTCCTACCAGAGACACGTAG
 C  R  R  D  Y  L  R  L  F  G  Q  D  G  L  C  A  S>

       310       320       330       340       350
CTGTGACAAGCGGATCCGTGCCTATGAGATGACGATGCGGGTGAAAGACA
GACACTGTTCGCCTAGGCACGGATACTCTACTGCTACGCCCACTTTCTGT
  C  D  K  R  I  R  A  Y  E  M  T  M  R  V  K  D>

       360       370       380       390       400
AAGTGTATCACCTGGAGTGTTTCAAATGCGCCGCCTGTCAGAAGCATTTC
TTCACATAGTGGACCTCACAAAGTTTACGCGGCGGACAGTCTTCGTAAAG
K  V  Y  H  L  E  C  F  K  C  A  A  C  Q  K  H  F>

       410       420       430       440       450
TGTGTAGGTGACAGATACCTTCTCATCAACTCCGACATAGTGTGTGAACA
ACACATCCACTGTCTATGGAAGAGTAGTTGAGGCTGTATCACACACTTGT
 C  V  G  D  R  Y  L  L  I  N  S  D  I  V  C  E  Q>
```

```
        460       470       480       490
AGACATCTACGAGTGGACCAAGATCAATGGGATCATCTAG
TCTGTAGATGCTCACCTGGTTCTAGTTACCCTAGTAGATC
  D  I  Y  E  W  T  K  I  N  G  I  I  *
```
